# Mode of Action and Specificity of *Bacillus thuringiensis* Toxins in the Control of Caterpillars and Stink Bugs in Soybean Culture

**DOI:** 10.1155/2014/135675

**Published:** 2014-01-20

**Authors:** Rogério Schünemann, Neiva Knaak, Lidia Mariana Fiuza

**Affiliations:** Programa de Pós-Graduação em Biologia—Laboratório de Microbiologia e Toxicologia, Centro de Ciências da Saúde, Universidade do Vale do Rio dos Sinos, Avenida Unisinos, 950, 93022-000 São Leopoldo, RS, Brazil

## Abstract

The bacterium *Bacillus thuringiensis* (*Bt*) produces delta-endotoxins that possess toxic properties and can be used as biopesticides, as well as a source of genes for the construction of transgenic plants resistant to insects. In Brazil, the introduction of *Bt* soybean with insecticidal properties to the velvetbean caterpillar, the main insect pest of soybean, has been seen a promising tool in the management of these agroecosystems. However, the increase in stink bug populations in this culture, in various regions of the country, which are not susceptible to the existing genetically modified plants, requires application of chemicals that damage the environment. Little is known about the actual toxicity of *Bt* to Hemiptera, since these insects present sucking mouthparts, which hamper toxicity assays with artificial diets containing toxins of this bacterium. In recent studies of cytotoxicity with the gut of different hemipterans, susceptibility in the mechanism of action of delta-endotoxins has been demonstrated, which can generate promising subsidies for the control of these insect pests in soybean. This paper aims to review the studies related to the selection, application and mode of action of *Bt* in the biological control of the major pest of soybean, *Anticarsia gemmatalis*, and an analysis of advances in research on the use of *Bt* for control hemipterans.

## 1. Introduction

Soybean, *Glycine max* (L.), is the largest agricultural commodity of economic importance in Brazil, occupying large areas of planting, targeting both domestic consumption and the export market. Given its economic importance, the problems caused by the attack of insect pests reduce production and decrease the quality of the grains or seeds [1]. Among the insect groups stands out the velvetbean caterpillar: *Anticarsia gemmatalis* (Hübner 1818), Lepidoptera: Noctuidae; the brown stink bug: *Euschistus heros* (Fabricius 1798), Hemiptera: Pentatomidae; the small green stink bug: *Piezodorus guildinii* (Westwood 1837), Hemiptera: Pentatomidae; and the green stink bug: *Nezara viridula* (Linneus 1758), Hemiptera: Pentatomidae.

The use of microorganisms has assumed a prominent position among the options that seek to control insect pests without the use of chemicals and with high specific toxicity applied in agroecosystems. The Gram-positive bacterium *Bacillus thuringiensis* (Berliner 1909), *Bt*, stands out representing approximately 95% of microorganisms used in biological control of agricultural pests in different cultures [2]. Besides the economic aspect and the safety to human health [3], this bacterium is the most promising for the production of biopesticides and plant resistant to insects, associated with environmental preservation [4].

Considering the application of *Bt* in the biological control of insects, Hansen and Salamitou [5] reported that the application of this entomopathogen is estimated at 13,000 tons of commercial formulated in the world, mainly applied against 2 Lepidoptera. Currently the largest single market for European biopesticides is Spain, followed by Italy and France. Despite that the overall market growth of biopesticides does not meet the expectations of the 1990s, the potential remains high and there are opportunities that could increase the total market in 2020 [6].

The efficiency of the use of this entomapathogen is characterized by the production, during the sporulation, of crystalline protein inclusions which are toxic to various insect groups. These proteins are produced in the form of protoxins and transformed into toxic peptides by a number of events which occur in the midgut after ingestion of *Bt*, killing the susceptible insect [7].

*B. thuringiensis* can also serve as a source of toxic genes that can be expressed in plants and thus confer toxic property against different species of insect pests. Genetically modified plants (GMPs) that express the *Bt* genes, such as rice, corn, potato, cotton, and soybean are associated with the control of pests, especially Lepidoptera. The resistant cultivars result in increased productivity, greater economic value, reduction in the use of chemical pesticides, and benefits in the selectivity of the target pest [4, 8]. Moreover, *Bt* plants favor the exposure of target insects throughout infestation period, including pests in their most vulnerable stages of development. In another instance, the specificity of toxins of this bacterium is effective for only a limited number of species and the development of resistance to modified plants is more specific than other insecticides [9].

The main pest, the velvetbean caterpillar, is a defoliator insect that can cause complete destruction of the plant [10, 11]. In some moments in the development of culture, as in phase between flowering and pod production, the attacked plants are able to withstand only 30% of defoliation caused by this insect, without incurring losses in seed production [12] Chewing insects are susceptible to control with *Bt* insecticides when applied on the surface of the leaves or when genes are inserted into the plant genome, such as in the case of soybean. Other insect pests, such as the hemipterans, being endophagous, have no larval stage and her nymphs and adults have sucking mouthparts, not favoring the ingestion of the microorganism.

Stink bugs are insects that come to prominence on soybean because the damage caused by these pests has caused a reduction in productivity. In the USA, in 2008, losses reached US$ 13 million [13]. The values of the losses caused by these pentatomids in Brazil are still difficult to be calculated, because they do not present dispersion throughout the national territory, being featured only in isolated regions. The emergence of large populations has been causing concern to farmers, as this pest complex is considered critical because it causes major impacts on key crops worldwide [14].

The lack of new technologies for the control of stink bugs is also worrying due to the increase of the indiscriminate use of insecticides for the direct control of these insects. Besides resistance to chemicals, we can also consider that the increased use of transgenic plant varieties resistant to primary pests has caused a decline in the application of chemical pesticides which in turn favors secondary pests such as stink bugs.

An alternative to the control of these sucking pests is the use of resistant cultivars expressing transgenic *Bt* genes, source of toxicity for the control of caterpillars. However little is known about the effect of *Bt* toxins to pentatomids.

## 2. *Bacillus thuringiensis*

*Bt* is an aerobic or anaerobic facultative and sporulating bacterium. It can remain latent in the environment even in adverse conditions for its development. *Bt* can be found in soil, insects and their habitats, stored products, plants, forest, and aquatic environments [15–18].

**Figure 1:**
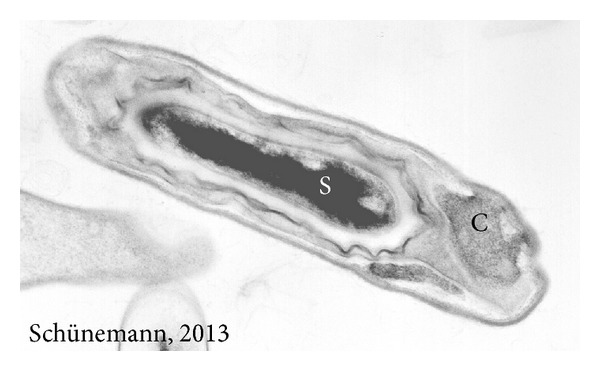
Transmission electron microscopy of *Bacillus thuringiensis*, spore (S), and crystal (C). Magnification 40.000x.

This bacterium differs from other species belonging to this genus by the presence of a parasporal inclusion body (crystal) of protein origin, formed during sporulation [19] (Figure 1). This crystal is composed of Cry proteins which are encoded by *Cry* genes [20, 21].

The crystals of bipyramidal, cuboid, rhomboid, oval, spherical, or even no definite shape [22] of a strain of *Bt* toxins may contain up to five different protein whit different molecular weight may vary between 30 and 142 kDa as in the case of strain HD-1 (*B. thuringiensis* subs. *Kurstaki*) which produces toxins Cry1Aa, Cry2Aa, Cry1Ab, and Cry1Ac Cry2Aa.

Cyt and Cry proteins that form the crystal, with toxic properties to insects, are soluble in water and belong to the *δ*-endotoxin class of bacterial proteins. Besides these, *Bt* also produces several other toxins such as *α*-exotoxin, *β*-exotoxin, hemolysins, enterotoxins, phospholipases, and chitinase [5].

The *δ*-endotoxins form two classes of toxins, the first group: *α*-helix toxins or *α*-helical (group of proteins that includes the Cry proteins containing three domains) to which the *α*-helix region of the protein forms a pore in the membrane and the second group: the *β*-barrel toxins (includes Cyt proteins), these are inserted into the membrane to form a *β*-barrel composed of *β*sheet hairpins from each monomer [23].

According to Monnerat and Bravo [24], the amino acid sequences, when aligned, allow the analysis of similarities between the Cry protein classes, revealing the presence of five blocks of conserved sequences located in the internal regions of the protein and in the contact region between domains. In some of these proteins, the blocks vary their position along the amino acid sequence, whereas others may be completely absent [19].

The relevance of Cry proteins is due to their toxic properties produced after ingestion by insects of different orders [25] and tie order of the toxicity is: Lepidoptera, Diptera, Coleoptera, Hymenoptera, Hemiptera, Isoptera, Orthoptera, Siphonoptera, and Thisanoptera. In the database of *Bt* ([26]: http://www.lifesci.sussex.ac.uk/home/Neil_Crickmore/Bt), 633 families and subfamilies of Cry proteins with toxic activity including mites, protozoa, and nematodes are described [20, 27–29].

Genes that express the delta-endotoxins are called “*cry genes*” due to the crystalline phenotype. These genes are located on plasmids of large molecular weight. Currently, due to their importance, more than 70 classes of *Cry* genes are described (cry1 the cry70). These endotoxins have been classified as Cry1–Cry69 and Cyt1–Cyt3 and different subgroups depending on their amino acid sequence (http://www.lifesci.sussex.ac.uk/home/Neil_Crickmore/Bt/), [26].

Of these, some *Bt* genes such as *cry1Ab*, *cry1Ac*, *cry2Ab*, and *cry9C* are already being commercially used in GMP such as corn, potato, soybean, and cotton in order to protect against lepidopteran pests of these crops, making it an alternative in reducing the application of chemical pesticides since 1996 [30]. The growth scenario of technology adoption could not be different in Brazil, the second in the world in area planted with GMP. In 2011, 160 million hectares were cultivated with Transgenic plants, with a growth of 12 million hectares compared to 2010, ensuring its place in the ranking of countries that use the technology of genetically modified organisms, behind only the United States [31].

The characterization of *Cry* genes is important for the differentiation of specific toxicity of Cry proteins active against certain orders of insects. For example, *cry1* group has several subclasses identified, each of which is responsible for a specific form of activity against various species of insects [4, 15, 23, 24, 32]. It is widely used in Lepidoptera, whose order focuses most of the studies on the mode of application and action. For some insect groups, such as dipterans or coleopterans, toxicity studies showed that the insecticidal activity of *Bt* has little relevance [33, 34].

## 3. Toxicity

Considering the ecology of *Bt*, studies performed by Aronson and Shai [56] showed that this microorganism may have a symbiotic relationship with plants,which perhaps explains the production of toxins so specific and efficient against insect pests. However, in the natural environment,several studies indicate that isolates without insecticidal activity are more widely distributed (over 90%) than those with toxic properties [57, 58].

Currently the demand for new *Bt* strains will increase the number of toxins available for pest control and management of their resistance [59]. Several isolates have been tested and characterized against insect pests and disease vectors to be used as basis for production of biopesticides or as donors of genes encoding insecticidal proteins [60].

In Lepidoptera, toxins are consumed through ingestion. *Bt* toxins classified and studied with insecticidal activity in this group are Cry1, Cry2, Cry9, and Cry15. However only the toxins of the Cry1, Cry2, and Cry9 groups were reported with insecticidal activity to *A. gemmatalis* (Table 1).

Regarding stink bugs, benefits from the development of an efficient system of integrated management of these pests by soybean farmers can only be achieved if there is improved information on these species, qualitatively and quantitatively.

Agricultural pests of the order Hemiptera, with mouthparts that penetrate and suck, at first, are not able to ingest the insecticide *Bt* Cry toxins expressed in GMP. Experiments have shown that only biting insects, mites, and thrips [61] are able to ingest the Cry1Ac protein from *Bt* plants, excluding the sucking pests [62, 63].

Monitoring nontarget insects of *Bt* cotton plants expressing Cry1Ac, Torres and Ruberson [61] suggested that feeding by herbivorous predators of insects susceptible to *Bt* toxin does not imply the development of *Podisus maculiventris* (Hemiptera: Pentatomidae). It suggests that the cultivation of *Bt* cotton provides an opportunity for the conservation of these predators in these ecosystems, which does not necessarily occur if other Cry proteins are used.

In studies, it has been proven that there is a certain susceptibility by some insects of the order Hemiptera to *Bt* toxins (Table 2). Many studies have investigated the potential effect of Cry toxins on nontarget arthropods in *Bt* plants [35, 64–67].

In some cultures, the population of secondary arthropods, which are not the target of *Bt* application, may be influenced by the implementation of these insecticidal properties [65]. In other agroecosystems, pentatomids of the genus *Podisus* are predators often used as biological control agents of defoliating caterpillars within the integrated pest management in agriculture and forestry systems already using *Bt* toxins. Of these, we highlight *P. maculiventris* (Say) (Hemiptera: Pentatomidae), which is probably the most important species in Europe and the United States and *P. nigrispinus* (Dallas) (Hemiptera: Pentatomidae) as the main predator species in plantations in Central and South America [68]. Therefore, the improvement and the detailed study of the specificity of *Bt* application become important in the management and preservation of the local ecosystem.

Brandt et al. [35] evaluated the proteolytic processing of the Cry1Ac and Cry2Ab toxins after their ingestion by *Lygus hesperus* Knight (Hemiptera: Miridae) and found the presence of proteolytic processing of toxins into the insect's digestive system. Porcar et al. [44], suggested a *Bt* strain with insecticidal activity against the three species of this order that are pests of conifers. Walters and English [43] and Porcar et al. [45] suggested toxic activity of isolates containing *cry2*, *cry3A*, *cry4Aa*, and *cry11Aa* genes against the potato aphid, *Macrosiphum euphorbiae* (Thomas) (Homoptera: Aphididae), and high toxicity to the pea aphid, *Acyrthosiphon pisum* (Harris) (Hemiptera: Aphididae), showing 100% mortality after feeding cith 500 g/mL or Cry4 or Cry11 proteins. In recent histochemistry studies with aphids, Li et al. [46] found that Cry1Ac toxins were hydrolyzed in the membrane of the stomach by cysteine proteases (CP), whereas Cry3Aa toxins were incompletely processed and partially degraded.

The association of Cry toxins with specific tissues studied by Brandt et al. [35] demonstrated that following ingestion of the Cry1Ac toxin activated with trypsin, the hemipteran *L. hesperus* has no receptor to this protein, but a wide link to in the microvilli of intestinal cells throughout the intestine for the Cry2Ab toxin.

The low susceptibility of these insects to *Bt* toxins may be related to similarities between the glycoproteins from insect midgut microvilli and not as a result of direct selection for toxicity, as shown by Li et al. [46]. *In vivo* assays showed that the toxins used showed low activity against the aphids, which may be related to the way the experiments were performed. Once nonsolubilized *Bt* crystals have been used in feeding assays, which present a disadvantage since the solubilization of the toxin does not occur due to the acidic pH in the stomach of these insects [69]. The proteolytic activation of the *Bt* toxin ingested in the gut of insects is essential to present toxicity. The differences between the proteolytic enzymes (abundance, type, and its correlation) and the pH of the midgut of hemipterans, as well as other pests, are factors that contribute to toxicity [46].

The intramolecular proteolytic cleavage is also important for toxicity against insects which have gut with acid or neutral pH, as this favors the creation of *Bt* plants that express Cry toxins instead of protoxins. The intramolecular proteolytic cleavage increases the solubility of the toxin in the gut, thereby facilitating its binding. These polypeptides can also associate with others and maintain the insecticidal activity [14]. This phase has been extensively studied using Cry1 toxins that involves removal of 27–29 N-terminal amino acids 500–600 and C-terminal amino acids. Hemipterans present acid gut and the proteolytic activities of the membrane are associated with cathepsin of the types G and B [69, 70].

More detailed studies carried out by da Cunha et al. [47], with the same insect, showed that the Cry1Ac toxin expressed in this plant generated humoral and cellular changes as a sign of response to a xenobiotic. The toxin ingested by a lepidopteran (third trophic level) induced changes in the distribution of glycogen, lipids, and calcium due to the disorganization of the perimicrovilllar matrix of the gut.

## 4. Histology and Mode of Action

Histological studies, using the insects gut, have been the focus of researches to control agricultural pests. This is due the fact that changes in the gut can not only affect their development, but also cause major physiological events, such as changes in nutrient absorption, degenerative transformation, appetite loss and abandonment of food, gut paralysis, physiological disorders, and total paralysis. These are the most common symptoms observed from the moment the susceptible insects ingest the *Bt* spores and crystals, leading to insect death, when larvae show a blackened color, a characteristic symptom of infections caused by this microorganism [24].

In lepidopterans, the chewing mouthparts promote the ingestion of *Bt* toxins both in a product form and in the form of a toxin-containing GMP. Their digestive tract is divided into three regions: preintestine (front), midgut, and hindgut and is one of the most important areas of contact between the insect and the environment. This has been the subject of research aiming to develop alternative control methods [71].

In caterpillars, the midgut epithelium, pseudostratified columnar, consists of four types of cells, which are involved in the processes of absorption and secretion of enzymes, which are columnar and caliciform cells with ionic homeostasis function, endocrine cells with endocrine function, and regenerative cells involved in the renewal of the epithelium [71]. All of them are coated by a peritrophic membrane, which serves to protect the epithelium from mechanical damage and also as a barrier against harmful chemicals and toxins.

According to Levy et al. [71], the columnar cells are in larger quantities, tall, and present long and numerous microvilli on the apical portion. The basal portion, invaginations form the basal labyrinth. The caliciform cells have a large central cavity delimitated by cytoplasmic projections filled with mitochondria. The regenerative cells present an electron-dense cytoplasm and few organelles. The endocrine cells are characterized by the presence of electron-dense secretory granules concentrated in the cytoplasm of the basal cell.

**Figure 2:**
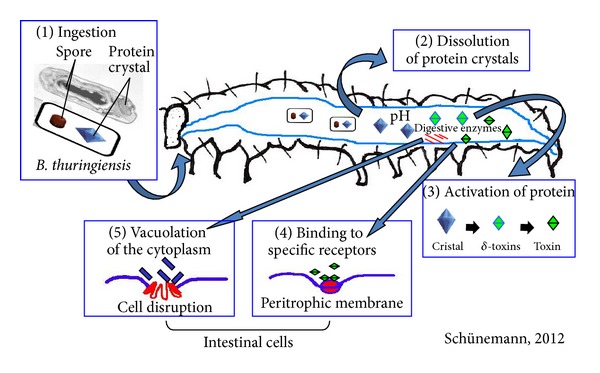
Mode of action of *Bacillus thuringiensis* in Lepidoptera: ingestion of bacteria (1); solubilization of the crystals (2); activation protein (3); binding of proteins to the receptors (4); membrane pore formation and cell lise (5).

Several histopathological and ultrastructural studies have investigated the interaction between *Bt* toxins in the midgut of larvae of these insects [72–77]. In Figure 2, the series of events that occur after ingestion of *Bt* in Lepidoptera are shown.

After ingestion, the crystals are solubilized in the alkaline (pH 9 to 12) midgut environment [78]. The proteins specific for Lepidoptera are soluble at pH above 9.5 [79]. The pH of the midgut of insects has great influence on the specific activity of Cry toxins. Some toxins are activated under alkaline conditions (CryII1A) and others are activated under conditions of neutral to acid pH (Cry1b) [23]. Cleavage of Cry toxins is a crucial step in the activation of the toxin and also in its specificity in different insects.

When toxins are solubilized, protoxins are released through the action of proteases resulting in active proteins of 60–70 kDa [80]. Their toxic fragments were described as connected to the N-terminal region of the polypeptide chain, which is later removed [81].

The protoxins are activated by digestive enzymes in the midgut and bind to specific receptors in the microvilli of the apical membranes of the columnar cells of the lepidopterans gut [19]. The binding of Cry toxins to the apical microvillus of the membrane vesicles of the insect determines the specificity of the Cry toxins [23].

Different proteins have been identified as receptors for *Bt* in Lepidoptera, and we can highlight with Lepidptera, including aminopeptidases [82], “cadherin-like protein” [83], and alkaline phosphatase [84].

The binding method may be described as a biphasic step consisting of reversible and irreversible stage [85, 86]. The interaction between the toxin and its binding site is a basic requirement for the toxicity [20]. The degree of toxicity is determined when the insertion in the cell membrane is considered irreversible binding [24].

The molecules for binding of Cry proteins are a cadherin type protein (CADR), an aminopeptidase anchored to a glicosilfosfatidil-N-inositol (GPI), an alkaline phosphatase anchored to a glicosilfosfatidil-inositol (GPI), and a 270 kDa glycoconjugate [23, 87]. Other experiments have shown that glycolipids may also be involved as receptor molecules in some insects and nematodes [87]. Studies conducted by Zhang et al. [88] suggested that the toxicity may be related to G-protein-mediated apoptosis upon binding to the receptor.

The Cry toxins cross the peritrophic membrane by binding to specific receptors on the apical membranes of intestinal cells causing opening or pore formation followed by vacuolation of the cytoplasm by osmotic imbalance between the intracellular and extracellular environments and cell disruption. This destroys the microvilli, causing the insect to stop feeding, leading to its death [7, 23, 76, 89, 90].

A second model to describe the mode of action of Cry toxins has been proposed by Zhang et al. [88] and refers to signal transduction. Although being only studied in insect cells, it does not differ from the first model to the stage of binding the protein formed by cleavage of the protoxin in intestinal microvilli.

Regarding stink bugs, these present needle-like sucking mouthparts that are formed by two mandibular and two maxillary stylets and a narrow channel which injects saliva in the salivary plant tissue. The digestion of these insects is extra-oral, secreting saliva in the food, which digests sap proteins using proteases present in saliva [91]. After sucking predigested nutrients, they are completely digested by proteases in the gut for absorption of nutrients.

The midgut of Hemiptera varies in its morphology and functional activity due to dietary habits of these insects. It is divided into an anterior dilated region, a median tubular region, and posterior dilated one [92]. The anterior region is responsible for electrolyte balance and involves the transport of ions and water and digestion of carbohydrates and is an important location for the storage of lipids. The process of digestion enzyme secretion is generally observed in the middle and posterior regions. Histologically, the pentatomids midgut has a simple epithelial layer and consists of digestive cells and regenerative distributed of all throughout the midgut. The hemipterans have no peritrophic membrane, therefore they are unprotected by a chitin and protein membrane that prevents the action of pathogens and excretion of digestive enzymes [93].

## 5. Application of **Bt ** Technologies

### 5.1. **Bt ** Products

Despite the importance and viability of microbial agents for pest control, only 2% of the insecticides used worldwide are based on the application of biopesticides, in which *Bt* represents approximately 95% of microorganisms used [94]. For over 50 years, *Bt* has been used in formulations for the biological control of many agricultural pests and vectors of human diseases and based on more than 90% of commercially available microorganism products [95, 96].

Since the first product has been launched in France in 1938, over 100 formulations were placed in the world market, with the American continent being responsible for 50% of this market [97]. From this fraction, the United States and Canada account for 90% and the countries of Latin America ranking with only 8–10% of consumption concentrated, mainly in Cuba and Mexico, to control pests in cotton, banana, potato, citrus, vegetables, tobacco, corn, and pasture. In Brazil *Bt*-based products are used in approximately 150,000 acres [90] for the control of about 30 pests with agricultural importance.

There are different commercial *Bt* products developed for control agricultural insect pests and also against mosquito species. Most of the spore-crystal formulations are obtained from different strains. These include *B. thuringiensis* var. *kurstaki* (*Btk*)-isolate HD1 (contains Cry1Aa, Cry1Ab, Cry1Ac, and Cry2Aa proteins); *B. thuringiensis* var. *kurstaki* (*Btk*)-isolate HD73 (contains Cry1Ac); *B. thuringiensis* var. *aizawai*-isolate HD137 (contains Cry1Aa, Cry1B, Cry1Ca, and Cry1Da); *B. thuringiensis* var. San Diego and *B. thuringiensis* var. *tenebrionis*, (which contains Cry3Aa); *B. thuringiensis* var. *israelensis* (containing Cry4A, Cry4B, Cry11A, and Cyt1Aa) toxins.

### 5.2. *Bt* Plants

A selection of different soybean cultivars with resistance against lepidopterans and other insect classes. This occurs through genetic improvement of plants which express chemical characteristics that involve the production of toxins such as isoflavonoids, that act as repellents, feeding and oviposition suppressors, and digestibility reducers of these insects, is also a way to reduce the damage caused by these insects [98]. The use of these plants with other techniques of integrated pest management (IPM) can keep the pest population levels below the levels of economic damages [99].

The insecticidal effect of GMPs on natural enemies is a controversial topic. Even after rigorous risk assessment, planting these cultivars has aroused concern over environmental impacts such as gene escape and effects on biodiversity. Considering soybeans, the risk of horizontal gene transfer is remote because of the lack of compatible wild species. The gene escape to the conventional crops can occur but can be avoided by isolation of the cultures [100, 101].

Plants with insecticidal activity, since they are rapidly degradable, reduce environmental impact, in addition to maintaining the beneficial fauna [102]. Their resistance may contribute to the reduction of the insect population below the economic injury level, do not cause imbalances in agroecosystems, and have a cumulative and persistent effect. They do not promote increases in production costs and are compatible with other control tactics [103].

Transgenic soybean is the main culture that currently occupies the crops of GMPs in the world, occupying 47% of the 160 million hectares of GM crops currently grown in the area in 2011. The soybean transformation was first reported in 1988 [104, 105]. To date, the transformation is not yet considered routine due to the complexity of combining techniques for its transformation and regeneration [54]. Transgenic soybean is an important tool for achieving economic growth of this crop in Brazil; however, it is essential to know how to use this technology and associate it with other tools.

*Bt* soybean is the second largest group of GMPs within this culture [106]. The cultivars are characterized by expressing Cry toxins with insecticidal properties to groups of insect pests of this crop. The toxins are expressed by cloned *Cry* genes from *Bt* and hence comes the name of the cultivar: *Bt* soybean. In Table 3, there are the papers published with the transformation of *Bt* soybean.

There is a great concern in making *Bt* available in large areas, as it may lead to selection of resistant phenotypes to this toxin. Susceptibility studies of toxic proteins to the major target pests have become important to obtain susceptibility levels and determine the diagnostic doses for future monitoring of possible changes in susceptibility.

In Brazil, research is limited to selecting isolates through bioassays and the description of its *Cry* genes. In the case of soybean, the *Bt* soybean roundup ready 2yield (*Bt*RR2Y) that expresses the Cry1Ac protein is produced on a commercial scale.

## 6. Final Considerations and Future Prospects

There are increasingly records of *Bt* isolates showing toxic activity to some insect pests. But in the market, there are few toxins of this microorganism used in formulated products and GMPs and may quickly cause the emergence of resistant populations. This fact occurred in different populations of Lepidoptera exposed to Cry toxins [107].

It is clear that cultures that express *Bt* toxins have had a very important beneficial impact on global agriculture due to the reduction in the number of pests and hence the total application of chemical insecticides used for its control, as well as the final production.

The large-scale cultivation of genetically modified crops over several years may increase the selective pressure on the pest species, which may result in the development of resistance [108]. Resistance to insect control practices is a threat to development, implementation, and maintenance of IPM practices. The emergence of resistance is often related to the reduction of pest populations by the increased mortality or reduced fecundity in insects. Thus, when there are differences in survival or fecundity between the specimens that form the population of a pest species after use of Cry proteins, the selection of resistant insects to protection techniques recently applied may occur.

Therefore, when there is significant variability in insect population with any physiological or behavioral characteristics that contrast the protection to Cry proteins insecticides, it is formed an inevitable selection of adapted insect pests. Thus, it essential to observe proper practices in the integrated management (IPM) of this pest, avoiding a continuous selection of resistant specimens, reducing the frequency of resistance that may compromise the protection to crops by pests.

Due to the large potential that pest populations have to develop resistance, some strategies have already been implemented in other cultures. As an example, using vegetative insecticidal proteins (VIPs) and using genes from plants or animals which encode immunosuppressive proteins [109].

In the cultivation of *Bt* soybean, the most recommended technique is the use of refuge areas. This consists of partially planting non-*Bt* plants in the area of soybean containing *Bt* proteins. The importance is to keep population of the target pest without exposure to *Bt* crops and thus sensitive to the proteins. This way, pest individuals that develop in the refuge can mate with any resistant individuals that may have survived in the culture. This would convey susceptibility to *Bt* for future generations of the pest, generating resistant and susceptible individuals within the population, preserving the *Bt* technology.

**Figure 3:**
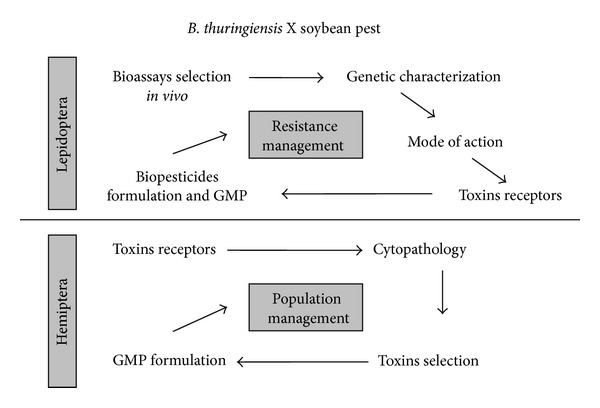
Comparative analysis of studies of the evolution of application of *Bacillus thuringiensis* to control caterpillars and stink bugs in agroecosystems.

Thus, assessing the mode of action of Cry toxins is a complex process that involves interaction with the receptor molecules that lead to different mechanisms of membrane insertion and lysis of intestinal cells. However the characterization of the mode of action of Cry toxins in other susceptible organisms may be important for understanding the mode of action of this protein family in different insects, which often do not show insecticidal activity with *in vivo* toxicity tests. The analysis of interaction between insects and bacteria is essential for effective management of the target species, and steps for evaluation may not be the same for both (Figure 3). Advanced studies provided detailed techniques in each step of interaction between insect and pathogen, which can reveal new ways of using *Bt* toxins.

However, there is no information about the digestive physiology of hemipterans associated with *Bt* toxins, and no literature regarding the mechanism of resistance or susceptibility of these insects to Cry toxins is available. Thus, in Brazil where these species have an agricultural importance, it is necessary to evaluate the *in vitro* action of Cry proteins in the gut of these insects, testing the hypothesis of degradation or activation of Cry toxins in the gut of these pentatomids.

This knowledge may be through the identification of novel molecules and the development of bioinsecticides. In this context, it is of great importance the discovery of bacterial strains with increased activity, adapted to environmental conditions and with high specificity. Thus, the mapping of new Cry proteins to nonsusceptible insects and specificities of the receptors may be critical for the development of new products for the control of these insects.

Studies on the identification of receptor molecules and binding sites, especially in Hemiptera and Lepidoptera, are important for the development of strategies to control insect resistance. In addition, knowledge of the target molecules for insecticides for agricultural pests is also essential to use in GMPs in order to control the different susceptible targets to the toxins used.

## Figures and Tables

**Table 1:** *Bacillus thuringiensis* genes with toxic activity against *Anticarsia gemmatalis* in South America.

Gene	kDa	CL_50_	Author/local
*cry1Ac, cry1D* *cry1Aa, cry1Ab, cry1Ac, cry1C, cry1D *	130 130	1.69 ng/cm^2^ 0.49 ng/cm^2^	Brandtet al. [35] (Brazil)
*cry1Aa, cry1Ac, cry1Ie cry2Ab* *cry1Aa, cry1Ac, cry1Ie cry2Ab* *cry1D, cry2Ad* *cry1Aa, cry1Ab, cry1Ac, cry2A *	130 e 65	1.146 · 10^−6^ 1.614 · 10^−6^ 4.624 · 10^−7^ 1.873 · 10^−6^	Silva et al. [32] (Brazil)
*cry1Aa, cry1Ab, cry1Ac, cry2e cry1B* *cry1Aa, cry1Ab, cry1Ac, cry2e cry1B* *cry1Aa, cry1Ab, cry1Ac, cry2e cry1B *	130 e 65 130 e 65 130 e 65	15.16 ng/cm^2^ 17.22 ng/cm^2^ 21.49 ng/cm^2^	Praça et al. [36] (Brazil)
cry1 cry1 cry1,* vip3A *		7 ppm 6.7 ppm 8 ppm	Franco-Rivera et al. [37] (Argentina)
*cry1Aa, cry1Ab, cry1Ac*	70 to 140	—	Ber$\overset{´}{\text{o}}$nand Salerno [38] (Argentina)
*cry1Ab, cry2* *cry1Aa, cry1B, cry2* *cry1Aa, cry1Ab, cry1Ac, cry1B, cry2* *cry1Aa, cry1Ab, cry1Ac, cry1Be cry2 *	130 e 65 130 e 65 130 e 65 130 e 65	5.1 ng/cm^2^ 0.21 ng/cm^2^ 3.3 ng/cm^2^ 13.7 ng/cm^2^	Monnerat et al. [39] (Brazil)
*cry9Bb, cry1I, vip3 *	131.4, 50, e 70	0.78 *μ*g/cm^2^ (*M. sexta*)	Silva-Werneck and Ellar [40] (Brazil)
cry 1	130	3.47–8.09 *μ*g/larva	Gobatto et al. [41] (Brazil)
*cry2Aa, cry2Ab, cry2Ace cry9A* *cry9A *	130, 90 e 45 70, 58 e 38	0.195 *μ*g/larva 0.191 *μ*g/larva	Fiuza et al. [42] (Brazil)

**Table 2:** Hemipterans susceptible to different *Bacillus thuringiensis* toxins.

Gene	Species/family	kDa	Author
*cry2*, *cry3A*, *cry4 *	*Macrosiphum euphorbiae* Aphididae		Walters and English [43]
*cry1Ac*	*Lygus hesperus *	*65 *	Brandt et al. [35]
*cry2Ab*	Miridae	*71 *	
*cry5Ac*	*Diprion pini *	58 to 155	Porcar et al. [44]
	Diprionidae		
*cry5Ba*	*Pristiphora abietina *	58 to 155	Porcar et al. [44]
	Tenthredinidae		
*cry3A*	*Acyrthosiphon pisum *	—	Porcar et al. [45]
*cry4Aa*	Aphidoidea		
*cry11Aa*			
*cry1Ac*			
*cry3Aa*		60 25 and 37	Li et al. [46]
	*Podisus nigrispinus *		da Cunha et al. [47]
	Pentatomidae		

**Table 3:** Papers published with the transformation of *Bt* Soybean.

Gene	Insects	Effects	References
*cry1Ab* native	*Anticarsia gemmatalis *	Prevention of larvae feeding and growth	Parrott et al. [48]
*cry1Ac *(*tic107*) synthetic MON87701 event (Protein TIC107)	*Anticarsia gemmatalis* *Pseudoplusia includens *	Tolerant to attack	Fischhoff and Perlak [49]
*cry1Ac* synthetic	*Anticarsia gemmatalis *	100% mortality to *A. gemmatalis *	Stewart Jr et al. [50]
*cry1Ac *synthetic	*Pseudoplusia includens* *Helicoverpa zea *	Decrease of feeding and survival	Walker et al. [51]
*cry1A *synthetic	*Anticarsia gemmatalis* *Pseudoplusia includens,* *Epinotia aporema* *Rachiplusia nu* *Spilosoma virginica *	Elimination of infestation in greenhouse	MacRae et al. [52]
*cry1A* synthetic	*Pseudoplusia includes* *Helicoverpa zea* *Anticarsia gemmatalis *	High level of resistance	Miklos et al. [53]
*cry1Ac* synthetic	*Anticarsia gemmatalis *	Highly toxic	Homrich et al. [54]
*cry1Ac* (*tic107*)	*Anticarsia gemmatalis* *Pseudoplusia includens* *Hypena scabra *	Elimination of infestation in greenhouse	McPherson and MacRae [55]
